# Tumor-binding antibodies and tumor immunity

**DOI:** 10.18632/oncotarget.4889

**Published:** 2015-07-17

**Authors:** Yaron Carmi, Edgar G. Engleman

**Affiliations:** School of Medicine, Department of Pathology, Stanford University, Palo Alto, CA, USA

The remarkable capacity of the immune system to distinguish self from non-self makes it almost impossible to transmit cancers (or organs) from one individual to another in the absence of immunosuppressive drugs. Whereas rejection of donor cells bearing mismatched MHC alleles has been ascribed to direct activation of T cells by the altered MHC-peptide structures, the factors that initiate rejection of MHC-matched donor cells remain elusive. Our recent study identified these factors [1]. We found that whereas tumor cells grow steadily in syngeneic hosts, they spontaneously regress following transfer to MHCI and II matched allogeneic hosts, in a T cell-dependent manner. In such hosts, tumor reactive T cells were found to proliferate in secondary lymphoid organs and infiltrate tumor sites only after 5-6 days. At that time, the tumors were already infiltrated with activated dendritic cells (DC) that had consumed tumor cells and were processing their antigens in a stimulatory context, as indicated by their expression of CD40 and CD86 and secretion of IL-12. By contrast, DC in syngeneic tumors did not have an activated phenotype or evidence of tumor uptake. Importantly, when DC from naïve animals were cultured with allogeneic or syngeneic tumor cells, they did not become activated or ingest tumor cells, indicating that other factors present in tumor-bearing hosts must play a role in these processes. Further investigation revealed that the improved DC activation observed in allogeneic hosts results from the actions of pre-existing naturally occurring IgG antibodies that bind tertiary protein structures on the surface of allogeneic tumor cells. Thus, the rapid (within hours) binding of injected tumor cells by these alloantibodies is a key element that enables tumor-infiltrating DC to process tumor antigens and present them in a stimulatory context to CD4^+^ T cells.

The discovery that natural antibodies initiate the rejection of allogeneic tumors led us to assess the impact of these antibodies on tumors that arise in the autologous setting. The effects of tumor-binding IgG during autologous tumor initiation and progression have been a source of controversy for many years. On the one hand, circulating antibodies against p53 are associated with poor prognosis and metastases in a variety of human cancers (reviewed in [2]). Moreover, in μMT mice that lack B cells, vaccination with irradiated tumor cells promoted a protective Th1-biased immune response, while vaccination of wild-type animals generated a poorly protective Th2-biased response [3]. In other studies tumor-binding IgG was shown to promote tumor progression and escape through various mechanisms including the induction of regulatory macrophages and release of proangiogenic and growth factors from mast cells (reviewed in [4]). On the other hand, these results contrast with studies showing that tumor-binding IgG can kill tumor cells, either directly by fixing complement, or by inducing antibody-dependent cell-mediated cytotoxicity (ADCC). Moreover, engagement of Fc receptors on DC by immune complexes (IC) is known to promote their activation and cross presentation of internalized antigens. Indeed, vaccination of mice with bone marrow DC (BMDC) loaded with ovalbumin-IgG IC can protect recipients from ova-expressing tumor challenge via anti-ova T cell-mediated immunity (reviewed in [5, 6]).

Nonetheless, in our study injections of allogeneic tumor-binding IgG into syngeneic tumor-bearing mice had little or no treatment effect. The discrepancy between the capacity of IC to induce DC activation in vitro, and the inability to apply this principle *in vivo* in the autologous tumor setting led us to a broad study of the interactions between various DC subsets and IC. The findings showed that while BMDC and splenic DC can efficiently process IC, tumor-associated DC are not able to internalize and process IC unless exposed to additional stimuli such as TNFα and CD40 ligand. Intratumoral injection of tumor-binding IgG in combination with such stimuli induced remarkably potent anti-tumor immunity that resulted in eradication of established tumor nodules and distant metastases through recruitment and activation of tumor-reactive T cells. This treatment proved effective in a number of mouse tumor models and despite its potency, no autoimmune disease or other serious toxicity was detected.

In subsequent studies, we used mass spectroscopy to identify the antigens on B16 melanoma recognized by the alloantibodies, and found no relationship between these antigens and those recognized by tumor-infiltrating T cells in successfully treated mice. For example, although none of the antibodies were directed against TRP-2 or gp100, large numbers of activated CD8 T cells recognizing these proteins were found in tumors injected with the alloantibodies. In agreement with this finding, Zhu et al. recently demonstrated a synergistic effect between anti-gp75 antibodies and CD8 T cells that recognize gp100 [7]. Our data also suggest that regardless of the antigens bound by anti-tumor antibodies, the resulting T cell response is directed mainly at tumor specific antigens, which may explain why the combination of anti-tumor antibodies and DC stimulation is so well tolerated. Taken together, these findings show that the impact of tumor-binding IgG on tumor growth is context dependent. In an immunostimulatory milieu, such antibodies can induce powerful anti-tumor immunity that can potentially be harnessed for the treatment of patients with cancer.
